# Melatonin mediates intestinal barrier dysfunction and systemic inflammation in moderate-severe OSA patients

**DOI:** 10.1080/07853890.2024.2361825

**Published:** 2024-07-08

**Authors:** Zhicheng Wei, Hangdong Shen, Fan Wang, Weijun Huang, Xinyi Li, Huajun Xu, Huaming Zhu, Jian Guan

**Affiliations:** a Department of Otorhinolaryngology Head and Neck Surgery, Shanghai Sixth People’s Hospital Affiliated to Shanghai Jiao Tong University School of Medicine, Shanghai, China; b Shanghai Key Laboratory of Sleep Disordered Breathing, Shanghai, China; c Otorhinolaryngology Institute of Shanghai Jiao Tong University, Shanghai, China

**Keywords:** Obstructive sleep apnoea, melatonin, intestinal barrier dysfunction, systemic inflammation, ZO-1, LPS, CRP

## Abstract

**Background:**

Intestinal barrier dysfunction and systemic inflammation are common in obstructive sleep apnoea (OSA). We aimed to investigate the role of melatonin, an anti-inflammatory mediator, in mediating the relationships between OSA, intestinal barrier dysfunction and systemic inflammation.

**Methods:**

Two hundred and thirty-five male participants who complained with sleep problems and underwent whole night polysomnography at our sleep centre between 2017 and 2018 were enrolled. Polysomnographic data, anthropometric measurements and biochemical indicators were collected. Serum melatonin, intestinal barrier function biomarker zonula occludens-1 (ZO-1) and inflammatory biomarkers C-reactive protein (CRP) with lipopolysaccharide (LPS) were detected. Spearman’s correlation analysis assessed the correlations between sleep parameters, melatonin and biomarkers (ZO-1, LPS and CRP). Mediation analysis explored the effect of OSA on intestinal barrier dysfunction and systemic inflammation in moderate-severe OSA patients.

**Results:**

As OSA severity increased, serum melatonin decreased, whereas ZO-1, LPS and CRP increased. Spearman’s correlation analysis showed that serum melatonin was significantly negatively correlated with ZO-1 (*r* = −0.19, *p* < .05) and LPS (*r* = −0.20, *p* < .05) in the moderate-OSA group; serum melatonin was significantly negatively correlated with ZO-1 (*r* = −0.46, *p* < .01), LPS (*r* = −0.35, *p* < .01) and CPR (*r* = −0.30, *p* < .05) in the severe-OSA group. Mediation analyses showed melatonin explain 36.12% and 35.38% of the effect of apnoea–hypopnea index (AHI) on ZO-1 and LPS in moderate to severe OSA patients.

**Conclusions:**

Our study revealed that melatonin may be involved in mediating intestinal barrier dysfunction and systemic inflammation in moderate-to-severe OSA patients.

## 1. Introduction

Obstructive sleep apnoea (OSA) is a common sleep-related breathing disorder that affects an estimated 936 million adults worldwide, with the largest number in Chinese [1]. It is mainly characterized by recurrent hypopnea and apnoea during sleep, including intermittent hypoxemia and sleep fragmentation (SF), which can lead to hypertension, coronary heart disease, diabetes, cerebrovascular diseases, dementia, mood disorders and even sudden death at night [2–6].

OSA characteristic pathological changes, chronic intermittent hypoxia (CIH) and SF, could induce oxidative stress and further lead to activation of proinflammatory cascade and systemic inflammation [7–11]. Thus, inflammatory biomarkers were increased in OSA patients, such as C-reactive protein (CRP) and lipopolysaccharide (LPS) [12–14]. The source of inflammatory biomarkers might be derived from gut intestinal leakage as evidenced by elevated zonula occludens-1 (ZO-1) [15]. Functions of the intestinal mucosal barrier and the intestinal microbiota are impaired in OSA patients, which are highly sensitive to hypoxia [16,17]. Previous studies confirmed that intestinal barrier biomarkers and inflammatory markers are associated with apnoea–hypopnea index (AHI) in middle-aged OSA patients [15,18].

Melatonin is a neurohormone synthesized mainly in the pineal gland of animals and is responsible for regulating the biological clock [19–22]. In addition to regulating day rhythm, melatonin is an important anti-inflammatory agent or antioxidant. The gut, with the help of the gut microbiota, can also synthesize melatonin and exert its antioxidant and anti-inflammatory effects through paracrine and autocrine pathways [23]. Melatonin has a positive protective effect against both endogenous irritation (gastric acid and pepsin) and exogenous damage (alcohol and stress) in the gastrointestinal tract [24]. Studies have found that OSA has a significant impact on the level of serum melatonin, which can cause the disturbance of the physiological secretion rhythm of melatonin in patients and reduce the level of serum melatonin in the morning [25]. In addition, melatonin has also been found in animal experiments to relieve high blood pressure and vascular endothelial dysfunction caused by intermittent hypoxia in rats [26]. It was also found that prophylactic use of melatonin in rats to reduce myocarditis and myocardial fibrosis as well as ischemia–reperfusion injury caused by CIH [27]. However, whether melatonin could mediate the relationships between OSA, intestinal barrier dysfunction and systemic inflammation was unknown.

In this study, we aimed to clarify the relationship among serum melatonin, intestinal barrier dysfunction and systemic inflammation in OSA.

## 2. Materials and methods

### 2.1. Study participants

A consecutive sample of all participants referred for suspected OSA and presented with snoring and/or daytime sleepiness, was recruited from our sleep centre between 2017 and 2018. All participants completed a survey that collected basic information such as health status and personal medical history. Written informed consent was obtained from all participants. The human study protocol was approved by the Ethics Committee of Sixth People’s Hospital Affiliated to Shanghai Jiao Tong University School of Medicine and conducted in accordance with the Declaration of Helsinki.

The exclusion criteria: (1) age less than 18 years; (2) female participants; (3) previous treatment for OSA (i.e. treatment with an oral appliance, surgery or continuous positive airway pressure); (4) taking melatonin medications; (5) treating with probiotics or prebiotics; (6) taking serotonin reuptake inhibitors; (7) suffering from cardiovascular and cerebrovascular diseases; (8) suffering from digestive system disease; (9) having an inflammatory or infectious disease; (10) suffering from rheumatic immune system disease; (11) suffering from benign and malignant tumours; (12) suffering from other sleep disorders; (13) other medical conditions such as severe trauma, fractures, reflex injuries; (14) recent vaccination or surgery; (15) use of glucose-lowering, lipid-lowering or antihypertensive drugs.

### 2.2. Sleep evaluation

To objectively evaluate sleep and breathing parameters, all participants underwent a comprehensive overnight laboratory-based polysomnographic monitoring (Alice 5, Philips Respironics Inc., Murrysville, PA). Skilled sleep technicians manually scored and collected various physiological data, including electroencephalographic, electrooculographic, chin electromyographic, electrocardiographic, rib cage and abdominal movement, oronasal airflow (measured via nasal pressure and oronasal thermistor), pulse oxygen saturation (SpO2), body position. These data were evaluated according to the 2012 criteria of the American Academy of Sleep Medicine [28].

Apnoea was defined as a decrease in nasal airflow of more than 90% from baseline that was sustained for at least 10 s, and hypopnea was defined as a decrease in nasal airflow of more than 30% associated with a decrease in oxygen saturation of 2.3%. AHI was defined as the number of apnoea and hypopnea events per hour during sleep; mean pulse oxygen saturation (MSpO_2_) was defined as the mean value of whole oxygen saturation observed during sleep; lowest pulse oxygen saturation (LspO_2_) was defined as the lowest value of whole oxygen saturation observed during sleep; oxygen desaturation index (ODI) was defined as the number of times per hour of sleep that the blood oxygen level dropped by ≥4% from baseline; microarousal index (MAI) was defined as the number of arousals per hour of sleep. Daytime sleepiness was assessed using the Chinese version of the self-administered Epworth Sleepiness Scale (ESS), scored on a range of 0–24, with higher scores indicating increased severity of sleepiness [29]. In accordance with the established guidelines of the American Academy of Sleep Medicine, OSA severity was categorized into four distinct levels: normal (AHI < 5), mild OSA (5 ≤ AHI < 15), moderate OSA (15 ≤ AHI < 30) and severe OSA (AHI ≥ 30) [30].

### 2.3. Anthropometric and biochemical measurements

Five anthropometric indices (i.e. height, weight, waist circumference (WC), neck circumference (NC) and hip circumference (HC)) were measured at baseline by trained physicians when wearing only undergarments and standing upright. Body mass index (BMI) was calculated as weight/height squared (kg/m^2^). Systolic blood pressure (SBP) and diastolic blood pressure (DBP) were measured to the nearest 2 mmHg using a mercury sphygmomanometer after participants had rested for 15 min. All of the basic parameters mentioned above were measured twice and mean values were calculated. All participants were requested to abstain from food and water after 10 p.m. on the night of PSG monitoring. Peripheral blood samples were obtained at 7 a.m. the next morning, and centrifuged at 3500 rpm for 10 min, after standing for 30 min at room temperature. Serum profiles, including fasting glucose, insulin, triglycerides (TGs), total cholesterol (TC), high-density lipoprotein cholesterol (HDL-C), low-density lipoprotein cholesterol (LDL-C), apolipoprotein A (ApoA) and apolipoprotein B (ApoB), were measured in the hospital’s clinical laboratory. The homeostatic model of insulin resistance (HOMA-IR) was calculated as glucose (mmol/L) × insulin (μU/mL)/22.5.

### 2.4. Detection of serum melatonin and biomarkers levels

In this study, ZO-1 was used to assess intestinal barrier dysfunction; CRP and LPS were used to assess systemic inflammation. Blood samples were taken from all set patients (*n* = 235) at 7 a.m. and allowed to stand for one hour at room temperature. Clear serum was clearly precipitated after one hour of standing in a cryogenic high speed centrifuge (Eppendorf, Hamburg, Germany) at 3000 rpm for 5 min. All serum samples were used for analysis of melatonin, ZO-1, LPS and CRP concentrations. The analyses were conducted using a competitive enzyme-linked immunosorbent assay (ELISA) (melatonin: CEA908Ge; ZO-1: SEC262Hu; CRP: SEA821Hu; LPS: IEB526Ge; USCN Life Science, Wuhan, China) following the manufacturer’s protocols. The intra-assay coefficient of variation was 7.9%. Each sample was tested in duplicate.

### 2.5. Statistical analysis

Normally distributed data are presented as means ± SDs, skewed data are presented as medians with IQRs, and categorical data are presented as percentages. Differences in baseline characteristics among the groups were examined using the Kruskal–Wallis *H* test, one-way analysis of variance (ANOVA) or the *χ*^2^ test according to the distribution characteristics of the data. SPSS software (ver. 22.0, SPSS Inc., Chicago, IL) was used to address most of the statistical analyses. Data are presented with descriptive statistics such as mean, standard deviation (SD), standard error of the mean (SEM) and percentage. Spearman’s rank correlation coefficients were calculated to determine the effect of PSG indices on serum variables and were displayed by a heatmap using the correlation plot model in OriginPro software (version 2021, OriginLab Inc., Northampton, MA). We applied the simple mediation model from the PROCESS macro in SPSS (model #4; version 2.16.3, IBM Corp., Armonk, NY) for mediation analysis, which included the bootstrapping procedure for bias-corrected bootstrap confidence intervals (CIs) [31]. Two-sided *p* values <.05 were considered significant.

## 3. Results

### 3.1. Basic characteristics

A total of 235 male subjects matched for age and BMI was finally included in our study, and the baseline characteristics are presented in Table 1. No significant differences in the WC, HC, waist/hip ratio, SBP and DBP were observed between the non-OSA group and OSA groups. Fasting glucose, insulin, HOMA-IR, TC, TG, HDL-C, LDL-C, ApoA and ApoB concentrations were no statistically significant differences between the groups. Compared with non-OSA group, severe-OSA group had lower MspO2 (*p* < .01) and LspO_2_ (*p* < .01), but had higher ODI (*p* < .01), MAI (*p* < .01) and ESS score (*p* < .05). Serum melatonin decreased gradually with increasing OSA severity. The serum melatonin concentration in moderate-to-severe OSA groups was significantly lower than that in the non-OSA group (all *p* < .05). In contrast, the levels of ZO-1, LPS and CRP progressively increased with OSA severity.

**Table 1.** Baseline characteristics of male subjects.

Variables	Non-OSA group	Mild-OSA group	Moderate-OSA group	Severe-OSA group
*Demographics*				
Number	60	59	55	61
Males (%)	100	100	100	100
Age (years)	41.27 ± 8.84	43.00 ± 8.99	42.81 ± 7.06	43.47 ± 8.12
Height (m)	1.74 ± 0.05	1.73 ± 0.05	1.73 ± 0.05	1.72 ± 0.06
Weight (kg)	78.54 ± 10.28	77.98 ± 9.09	75.29 ± 7.56	77.54 ± 10.82
BMI (kg/m^2^)	25.84 ± 2.75	25.96 ± 2.33	25.29 ± 2.48	26.00 ± 2.73
WC (cm)	93.00 ± 9.97	95.33 ± 7.74	95.69 ± 6.56	95.15 ± 7.36
HC (cm)	101.36 ± 6.23	101.28 ± 6.29	100.53 ± 4.56	100.74 ± 5.94
Waist/hip ratio	0.92 ± 0.07	0.92 ± 0.14	0.93 ± 0.44	0.94 ± 0.05
SBP (mmHg)	124.92 ± 12.53	125.65 ± 15.08	125.06 ± 13.39	126.57 ± 14.72
DBP (mmHg)	79.77 ± 8.79	82.65 ± 11.06	79.11 ± 11.37	83.92 ± 11.70
*Glucometabolic and lipometabolic indices*				
Fasting glucose (mmol/L)	5.47 ± 1.15	5.17 ± 0.62	5.33 ± 1.11	5.42 ± 0.83
Fasting insulin (μU/mL)	10.33 ± 6.08	11.08 ± 5.31	9.86 ± 4.54	13.46 ± 10.61
HOMA-IR	2.70 ± 2.58	2.62 ± 1.65	2.40 ± 1.42	3.32 ± 2.96
TC (mmol/L)	4.44 ± 0.85	4.53 ± 0.85	4.67 ± 0.72	4.91 ± 0.94
TG (mmol/L)	1.72 ± 1.62	1.58 ± 0.85	1.99 ± 2.04	2.05 ± 1.31
HDL-C (mmol/L)	1.02 ± 0.24	1.03 ± 0.19	0.99 ± 0.19	1.05 ± 0.26
LDL-C (mmol/L)	2.76 ± 0.72	2.82 ± 0.82	2.96 ± 0.59	3.04 ± 0.74
Apo A (g/L)	1.06 ± 0.20	0.99 ± 0.14	1.07 ± 0.23	1.10 ± 0.17
Apo B (g/L)	0.81 ± 0.17	0.84 ± 0.21	0.86 ± 0.17	0.91 ± 0.18
*Sleep data*				
AHI	2.70 ± 1.30	10.13 ± 3.09**	22.11 ± 4.12**	57.07 ± 14.35**
MSpO_2_ (%)	96.01 ± 1.10	95.89 ± 1.47	95.23 ± 1.35	88.43 ± 19.30**
LSpO_2_ (%)	90.67 ± 8.14	83.68 ± 2.29	84.51 ± 7.79	71.26 ± 11.42**
ODI	3.49 ± 3.20	9.99 ± 4.64**	20.69 ± 6.61**	54.42 ± 17.97**
MAI	18.06 ± 13.30	21.90 ± 16.32	19.89 ± 14.05	34.90 ± 18.10**
ESS	4.06 ± 3.01	7.55 ± 4.26	6.61 ± 4.78	9.35 ± 5.78*
*Serum biomarkers*				
Melatonin (pg/mL)	114.41 ± 32.78	112.99 ± 31.83	110.18 ± 35.71*	110.13 ± 40.56*
ZO-1 (ng/mL)	364.72 ± 191.14	371.83 ± 201.99	394.88 ± 192.10*	572.84 ± 923.11*
LPS (pg/mL)	1.49 ± 1.25	2.12 ± 1.48	2.21 ± 1.61*	2.54 ± 1.71**
CRP (ng/mL)	19.41 ± 16.99	20.13 ± 32.10	22.48 ± 16.89*	23.20 ± 14.65**

BMI: body mass index; WC: waist circumference; HC: hip circumference; SBP: systolic blood pressure; DBP: diastolic blood pressure; AHI: apnoea–hypopnea index; MSpO2: mean pulse oxygen saturation; LSpO2: lowest pulse oxygen saturation; ODI: oxygen desaturation index; MAI: micro-arousal index; ESS: Epworth Sleepiness Scale; HOMA-IR: homeostasis model of assessment for insulin resistance index; TC: total cholesterol; TG: total triglycerides; HDL-C: high-density lipoprotein cholesterol; LDL-C: low-density lipoprotein cholesterol; Apo A: apolipoprotein A; Apo B: apolipoprotein B; ZO-1: zonula occludens-1; LPS: lipopolysaccharide; CRP: C-reactive protein.

Normally distributed values are displayed as means ± SD. Differences were analysed by independent sample *t*-test between OSA of varying severities and non-OSA (mild OSA vs. non-OSA, moderate OSA vs. non-OSA, and severe OSA vs. non-OSA), and denoted as follows: *p < .05; **p < .01.

### 3.2. The relationships between sleep parameters, melatonin, intestinal barrier function biomarker (ZO-1) and inflammatory biomarkers (CRP and LPS)

**Figure 1.**
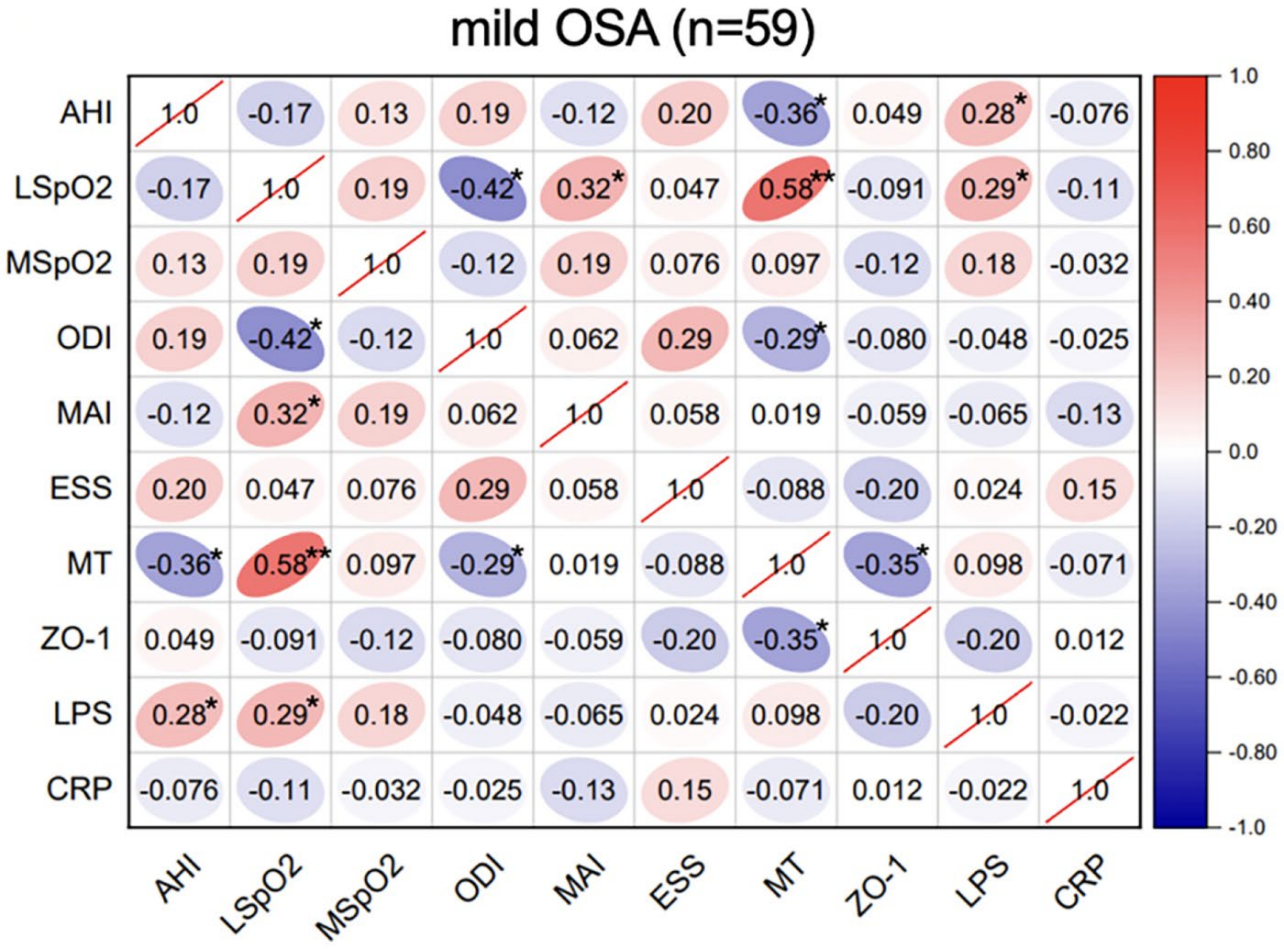
Correlations between sleep data and serum biomarkers in mild OSA. Spearman’s rank correlation coefficients were calculated to determine the influence of PSG variables on the levels of serum biomarkers in mild OSA (*n* = 59). The *r* values are represented by gradient colors, with red cells indicating positive correlations and the blue cells indicating negative correlations. *p < .05; **p < .01. AHI: apnea-hypopnea index; MSpO_2_: mean pulse oxygen saturation; LSpO_2_: lowest pulse oxygen saturation; ODI: oxygen desaturation index; MAI: micro-arousal index; ESS: Epworth sleepiness scale; MT: melatonin; ZO-1: zonula occludens-1; LPS: lipopolysaccharide; CRP: C-reactive protein; PSG: polysomnography.

**Figure 2.**
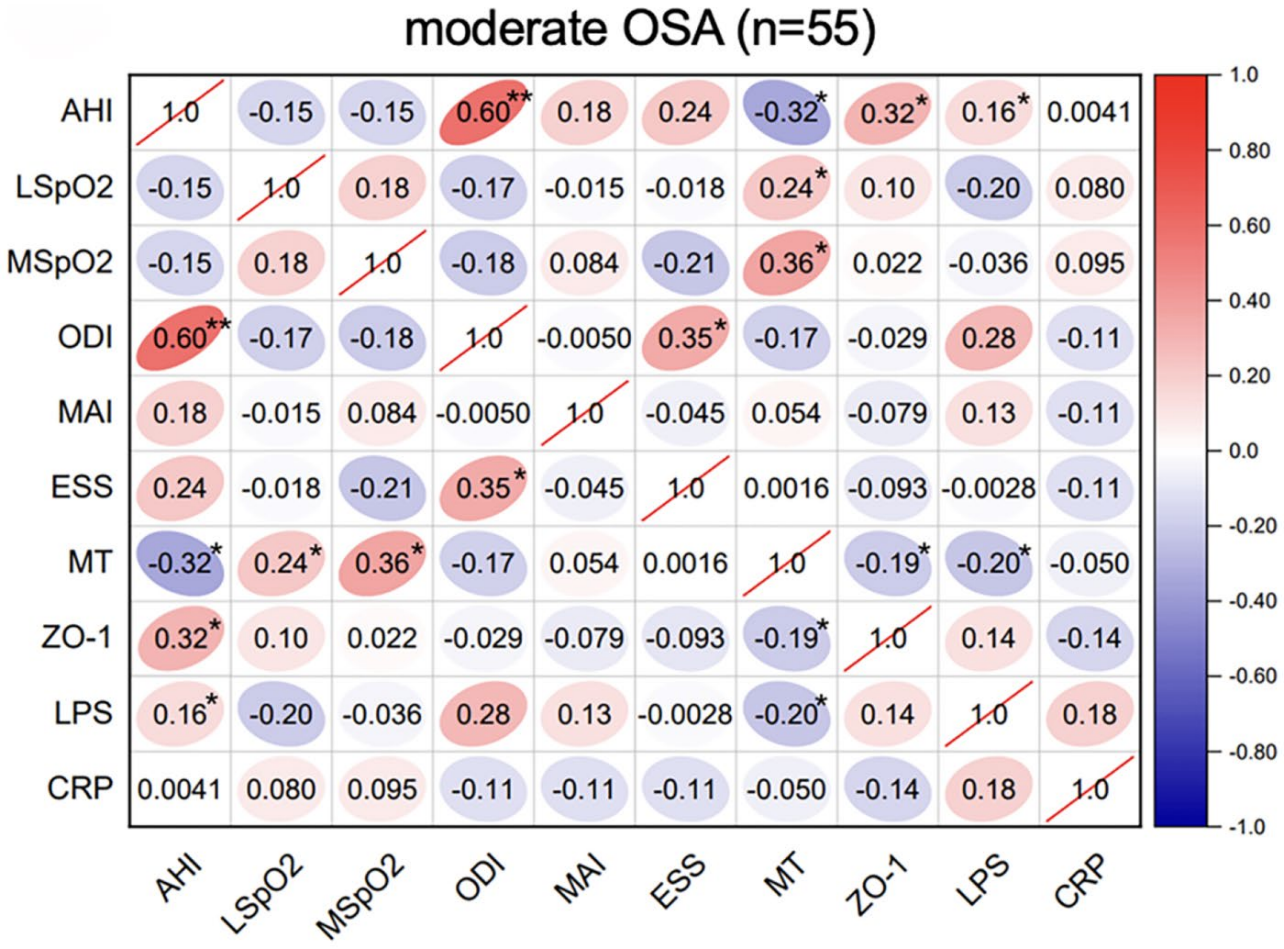
Correlations between sleep data and serum biomarkers in moderate OSA. Spearman’s rank correlation coefficients were calculated to determine the influence of PSG variables on the levels of serum biomarkers in moderate OSA (n = 55). The r values are represented by gradient colors, with red cells indicating positive correlations and the blue cells indicating negative correlations. *P < .05; **P < .01. Abbreviations: AHI: apnea-hypopnea index, MSpO2: mean pulse oxygen saturation, LSpO2: lowest pulse oxygen saturation, ODI: oxygen desaturation index, MAI: micro-arousal index, ESS: Epworth sleepiness scale, MT: melatonin, ZO-1: zonula occludens-1, LPS: lipopolysaccharide, CRP: C-reactive protein, PSG: polysomnography.

**Figure 3.**
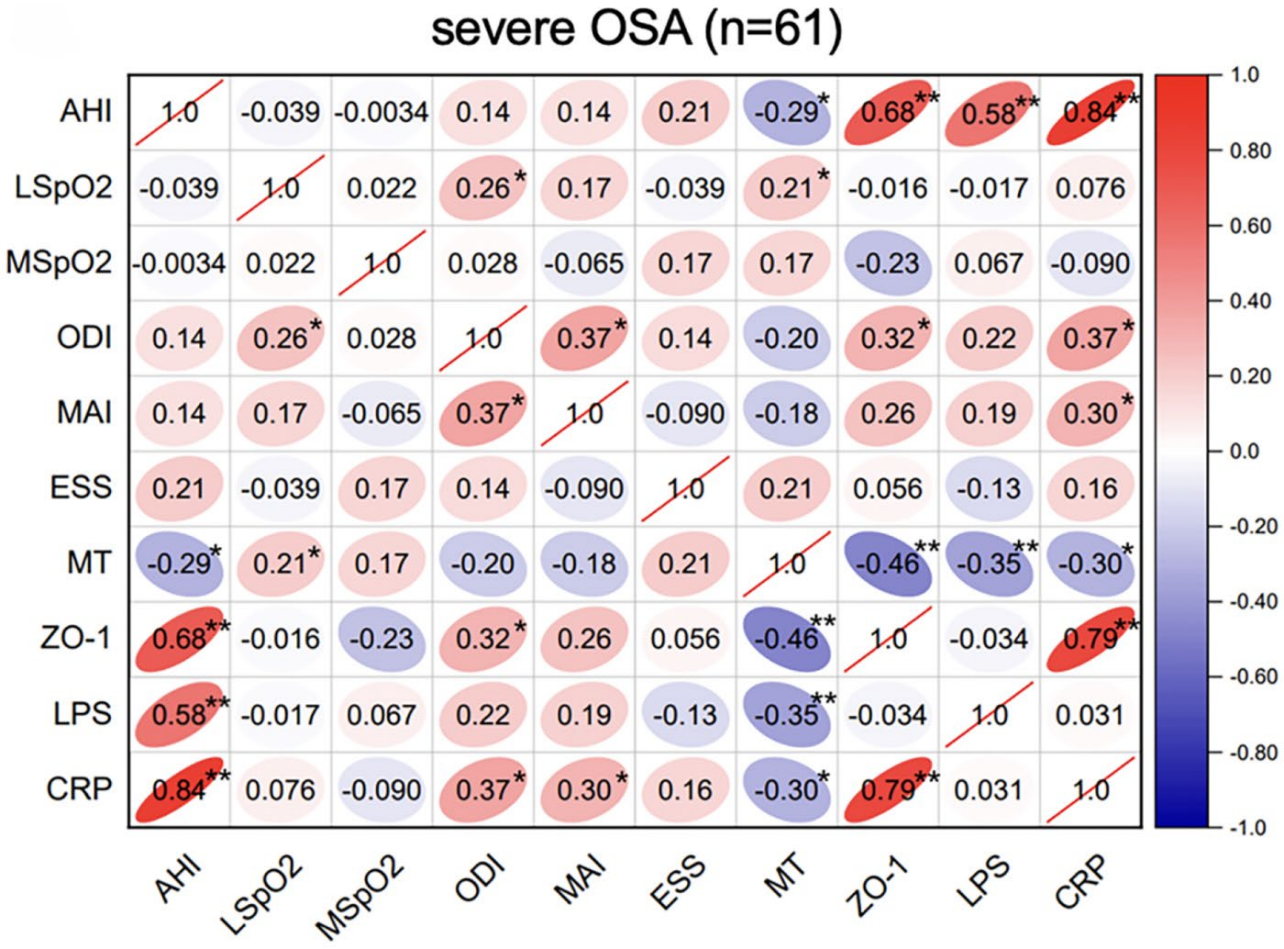
Correlations between sleep data and serum biomarkers in severe OSA. Spearman’s rank correlation coefficients were calculated to determine the influence of PSG variables on the levels of serum biomarkers in severe OSA (n = 61). The r values are represented by gradient colors, with red cells indicating positive correlations and the blue cells indicating negative correlations. *P < .05; **P < .01. Abbreviations: AHI: apnea-hypopnea index, MSpO2: mean pulse oxygen saturation, LSpO2: lowest pulse oxygen saturation, ODI: oxygen desaturation index, MAI: micro-arousal index, ESS: Epworth sleepiness scale, MT: melatonin, ZO-1: zonula occludens-1, LPS: lipopolysaccharide, CRP: C-reactive protein, PSG: polysomnography.

Spearman’s correlation analyses of the sleep parameters, serum melatonin, intestinal barrier function biomarker (ZO-1), inflammatory biomarkers (CRP and LPS) levels were performed in these OSA groups. In the mild-OSA group, melatonin was negatively correlated with the AHI (*r* = −0.36, *p* < .05) and ZO-1 level (*r* = −0.35, *p* < .05) (Figure 1). In moderate-OSA patients, melatonin was negatively correlated with the AHI (*r* = −0.32, *p* < .05), ZO-1 level (*r* = −0.19, *p* < .05) and LPS level (*r* = −0.20, *p* < .05). Additionally, ZO-1 (*r* = 0.32, *p* < .05) and LPS (*r* = 0.16, *p* < .05) levels were positively correlated with the AHI (Figure 2). In severe-OSA patients, melatonin showed negative correlations with the AHI (*r* = −0.29, *p* < .05), ZO-1 level (*r* = −0.46, *p* < .01), LPS level (*r* = −0.35, *p* < .01) and CRP level (*r* = −0.30, *p* < .05) (Figure 3).

In the severe-OSA group, AHI was positively correlated with the ZO-1 (*r* = 0.68, *p* < .01), LPS (*r* = 0.58, *p* < .01) and CRP (*r* = 0.84, *p* < .01). Furthermore, CRP showed a positive correlation with ZO-1 level (*r* = 0.79, *p* < .01) (Figure 3).

### 3.3. Melatonin mediated the relationship between OSA, intestinal barrier dysfunction and systemic inflammation

**Figure 4.**
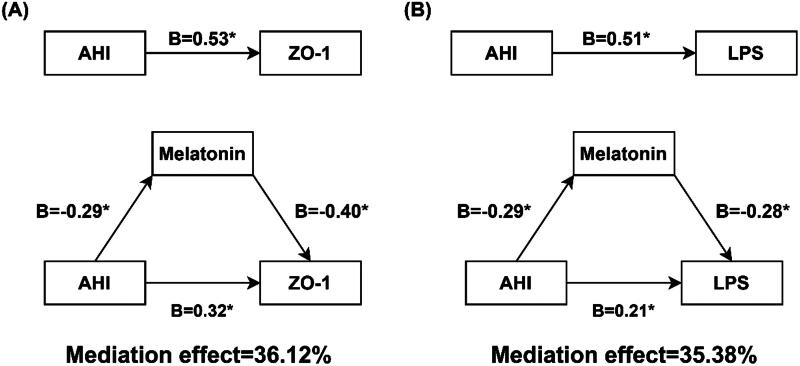
Path diagram showing how melatonin mediated the effect of AHI on intestinal barrier dysfunction and systemic inflammation in moderate-severe OSA patients. (A)shows melatonin mediates association between AHI and ZO-1; (B)shows melatonin mediates association between AHI and LPS. *means P< .05. B: the unstandardized coefficient. AHI: apnea-hypopnea index; ZO-1: zonula occludens-1, LPS: lipopolysaccharide.

**Figure 5.**
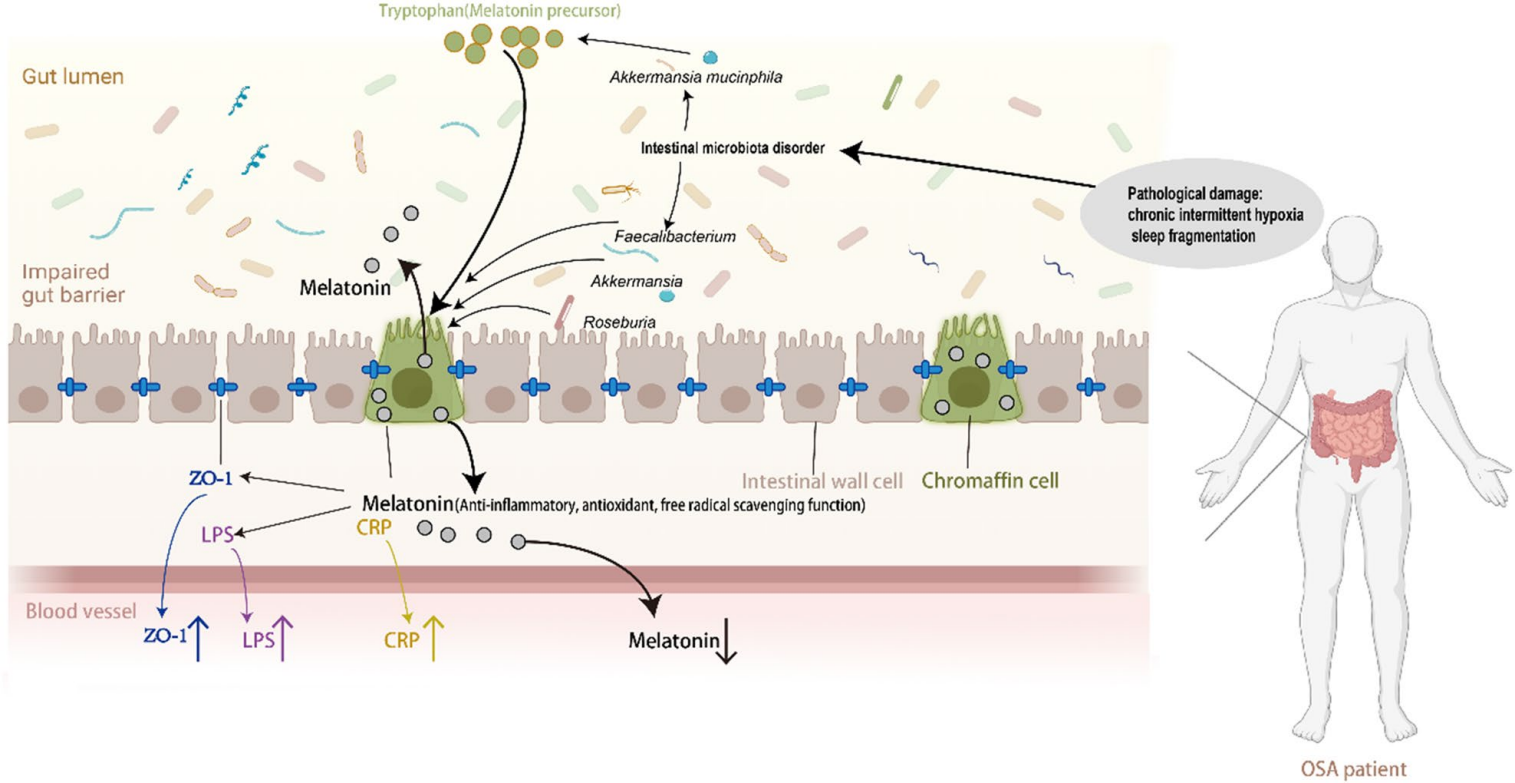
Schematic representation of the relationship between melatonin and intestinal barrier damage (biomarker: ZO-1) and systemic inflammation (biomarker: LPS, CRP). This figure was created using BioRender.com and further edited with Adobe Photoshop 2023 and Adobe Illustrator 2021. ZO-1: zonula occludens-1, LPS: lipopolysaccharide, CRP: C-reactive protein.

Next, we explored whether melatonin, as a mediating variable, mediated the effect of AHI on intestinal barrier dysfunction and systemic inflammation in moderate-severe OSA patients (*n* = 116, Tables S1–S3, S5–S7). Bootstrap mediation analysis of the relationship between AHI, ZO-1, and mediator melatonin yielded a significant intermediary effect (Tables S1 and S5 Figure 4(A)). Approximately, 36.12% of the AHI-generated gross effects was mediated via melatonin (*p* < .05). Likewise, bootstrap mediation analysis of the relationship between AHI, LPS and mediator melatonin also yielded a statistically significant intermediation at approximately 35.38% (*p* < .05) (Tables S2 and S6, Figure 4(B)) and 5).

## 4. Discussion

Female OSA patients are special, which are greatly affected by oestrogen and progesterone in the body [32]. Therefore, this study specifically targets middle-aged male patients as the population. In our study, we found that the serum melatonin from OSA patients decreased with the OSA severity, while ZO-1, LPS and CRP increased with it. Serum melatonin was significantly negatively correlated with ZO-1, LPS and CRP, and then mediated analysis determined that melatonin was a mediator in the relationship between AHI and ZO-1, LPS in moderate-to-severe OSA patients. It suggested that significant inhibition of melatonin in pathological states like intermittent hypoxia and SF, may be an important cause of intestinal barrier dysfunction and systemic inflammation in moderate-severe OSA patients.

As an anti-inflammatory substance, melatonin can alleviate inflammatory damage by inhibiting the production of inflammatory factors, clearing reactive oxygen species and free radicals, inhibiting NF-κB pathway, and reducing mitochondrial autophagy [33–35]. OSA patients are in a systemic inflammatory state and are often found to coexist with inflammatory diseases of various systems (including myocarditis, chronic kidney disease, irritable bowel syndrome, asthma, etc.) [7,36–41]. Consistent with previous studies, serum melatonin levels were significantly lower in OSA patients than in controls [25]. We found that levels of markers ZO-1, CRP and LPS were significantly negatively correlated with melatonin in OSA patients. The systemic inflammatory state of OSA may be highly related to the decrease of serum melatonin. A meta-analysis of clinical trials on the anti-inflammatory effect of melatonin showed that exogenous melatonin reduced the levels of a variety of inflammatory biomarkers, which may be helpful for the prevention and adjuvant treatment of inflammatory diseases [42]. The meta-analysis by Akbari et al. showed that melatonin had a favourable effect on reducing inflammatory markers such as CRP and IL-6 in patients with metabolic syndrome [43]. The relationship between melatonin and systemic inflammation and intestinal barrier dysfunction in OSA patients has not been previously reported. Distributed in the gastrointestinal tract, melatonin plays an important role in local antioxidant and intestinal motility regulation [44]. Our study demonstrated the mediating effect of serum melatonin in the influence of AHI on ZO-1 and LPS. These results suggest that exogenous melatonin supplementation in OSA patients may reduce intestinal barrier dysfunction and systemic inflammatory response. In fact, previous studies have reported the therapeutic effect of melatonin on intestinal mucosal injury and intestinal inflammatory diseases. Adjuvant melatonin therapy for ulcerative colitis can play an anti-inflammatory role and reduce the severity of ulcerative colitis [45]. In a sleep-deprived mouse model with colon mucosal damage and intestinal microbiome dysregulation, melatonin supplementation improved mucosal damage and colon microbiome dysregulation [46]. Melatonin is also an important hormone in the central nervous system to regulate circadian rhythm and sleep, and it is also an important target for the treatment of sleep rhythm disorder and insomnia in OSA patients. The melatonin agonist ramelteon can relieve the insomnia symptoms of OSA patients [47]. Long-term continuous positive airway pressure treatment can improve the circadian clock disorder caused by OSA and restore the secretion rhythm of melatonin and cortisol [48]. However, the improvement effect of melatonin on systemic inflammation and intestinal barrier damage in OSA patients and its mechanism need to be further studied, and melatonin is expected to become a new therapeutic drug [49–51].

It has been found that sleep deprivation interventions impair intestinal barrier function in rats by reducing blood melatonin levels through oxidative stress and activation of the NF-κB pathway [46]. Bertuglia and Reiter found that melatonin supplementation in hamsters alleviated oxidative stress and insulin resistance induced by CIH [52]. Some studies founded that the gut microbiome of OSA patients was significantly different from that of the normal population [37,38]. In our previous microbiota 16S rRNA analysis, it was found that the composition, abundance and metabolic function of intestinal microorganisms in mice with CIH model and SF model of OSA disease were significantly changed, and the relative abundance of *Clostridium* was significantly reduced [53]. Ten weeks of IH intervention caused changes in the abundance of certain bacteria in the gut (e.g. *Akkermansia muciniphila*, *Clostridium spp.*, *Lactococcus spp.* and *Bifidobacterium spp.*) in turn affected the levels of some metabolites, such as tryptophan (a synthetic precursor amino acid of melatonin), bile acids and branched-chain amino acids [53]. The gut microbiome contributes to melatonin synthesis in the colon, and the abundance of *Roseburia* was positively correlated with melatonin levels in the colon mucosa [54]. Melatonin can be recovered by supplementation with probiotics, including *Akkermansia* and *Faecalibacterium*, to improve intestinal microecological imbalance caused by sleep deprivation [55,56]. Our previous study found that exogenous melatonin supplementation could alleviate intestinal mucosal barrier damage, intestinal microbiota imbalance, intestinal Th17 polarization and systemic inflammation induced by CIH intervention in mice [57]. Previous studies have reported that OSA-related changes in intestinal microbiota may promote bacterial translocation through the defective intestinal barrier, which in turn promotes systemic inflammation [58]. It is speculated that the long-term pathological changes of OSA destroy the normal microbial environment and affect the synthesis of melatonin in the intestine. Under the chronic stimulation of intermittent hypoxia and SF, an abnormal microbiome and insufficient melatonin synthesis work together to cause damage to the intestinal barrier and systemic inflammation.

The main clinical treatment options for OSA are divided into non-surgical interventions (behavioural therapy, medical devices, etc.) and surgical interventions (uvulopalatopharyngoplasty, lateral pharyngoplasty, etc.), but few treatments can effectively alleviate the intestinal barrier dysfunction and systemic inflammation caused by OSA [59,60]. Our study offers promising prospects for the treatment of intestinal barrier dysfunction and systemic inflammation in OSA patients. This study is the first to investigate the relationship among melatonin and intestinal barrier function biomarker and systemic inflammation biomarkers in OSA patients, aiming to provide theoretical basis for the treatment of intestinal barrier function dysfunction and systemic inflammation in OSA patients. However, there are some limitations need to be discussed. First of all, due to the small number of female patients recruited, female patients were not included in our study, resulting in the lack of gender control in our study. Second, a relatively small OSA patients were enrolled. Third, confounding factors such as lifestyle (diet, exercise, smoking and alcohol consumption) were not considered. Fourth, only three characteristic biomarkers ZO-1, LPS and CRP, were selected, and other biomarkers were to be further studied. Fifth, this study was an exploratory cross-sectional study and could not effectively demonstrate a causal relationship between melatonin and intestinal barrier dysfunction and systemic inflammation. Sixth, further intervention experiments are needed to verify the effects of melatonin on intestinal barrier dysfunction and systemic inflammation in OSA patients. Deeper explorations are needed to determine the mechanism between melatonin, intestinal barrier dysfunction and systemic inflammation.

## 5. Conclusions

In our study, we explored the relationship between melatonin and intestinal barrier dysfunction and systemic inflammation in OSA patients, and found that melatonin may be involved in mediating intestinal barrier dysfunction and systemic inflammation in moderate-to-severe OSA patients.

## Supplementary Material

Supplemental Material

## Data Availability

The data that support the findings of this study are available from the corresponding author, [JG], upon reasonable request.
