# Supplementary material for: Melatonin mediates intestinal barrier dysfunction and systemic inflammation in moderate-severe OSA patients
Source: Ann Med. 2024 Jul 8;56(1):2361825. doi: 10.1080/07853890.2024.2361825 (PMC11232642; doi:10.1080/07853890.2024.2361825)
Supplement: Supplemental Material [file IANN_A_2361825_SM3004.docx]

Table S1. Mediation analysis: the role of melatonin as a mediator of AHI-associated effect on the ZO-1 level in patients with moderate-to-severe OSA.

| Regression analysis of mediation models (n=116) | | | | | | | | | | |
| --- | --- | --- | --- | --- | --- | --- | --- | --- | --- | --- |
| Variate | **Dependent variable: ZO-1** | |  | **Dependent variable: Melatonin** | |  | **Dependent variable: ZO-1** | | | |
|  | **β** | **t** |  | **β** | **t** |  | | **β** | **t** | |
| AHI | 0.53 | 13.28^*^ |  | -0.29 | 11.15^*^ |  | | 0.32 | 7.92^*^ | |
| Melatonin |  | |  |  | |  | | -0.40 | | 9.73^*^ |
| R^2^ | 0.60 | |  | 0.35 | |  | | 0.49 | | |
| F | 12.722^*^ | |  | 7.431^*^ | |  | | 2.763^*^ | | |

| Melatonin mediated the effects of AHI on ZO-1 level in patients with moderate-to-severe OSA | | | | |
| --- | --- | --- | --- | --- |
| Effect type | **Effect** | **BOOT SE** | **Bootstrap 95%CI** | |
|  |  |  | **LLCI** | **ULCI** |
| Gross effect | 0.897 | 0.071 | 0.758 | 1.036 |
| Direct effect | 0.573 | 0.077 | 0.421 | 0.724 |
| Indirect effect | 0.324 | 0.068 | 0.201 | 0.47 |
| Mediation effect | **36.120%** |  |  |  |

Positive regression coefficients (β) indicate positive effects. Negative regression coefficients (β) indicate negative effects. ^*^P < 0.05 or the 95% bootstrap confidence interval does not straddle zero. Abbreviations: AHI: apnea hypoxia index, ZO-1: zonula occludens-1, OSA: obstructive sleep apnea, SE: standard error, CI: confidence interval, LL: lower limit, UL: upper limit.

Table S2. Mediation analysis: the role of melatonin as a mediator of AHI-associated effect on the LPS level in patients with moderate-to-severe OSA.

| Regression analysis of mediation models (n=116) | | | | | | | | | | |
| --- | --- | --- | --- | --- | --- | --- | --- | --- | --- | --- |
| Variate | **Dependent variable: LPS** | |  | **Dependent variable: Melatonin** | |  | **Dependent variable: LPS** | | | |
|  | **β** | **t** |  | **β** | **t** |  | | **β** | **t** | |
| AHI | 0.51 | 8.26^*^ |  | -0.29 | 11.15^*^ |  | | 0.21 | 6.77^*^ | |
| Melatonin |  | |  |  | |  | | -0.28 | | 7.92^*^ |
| R^2^ | 0.47 | |  | 0.35 | |  | | 0.56 | | |
| F | 8.325^*^ | |  | 7.431^*^ | |  | | 1.066^*^ | | |

| Melatonin mediate the effects of AHI on LPS level in patients with moderate-to-severe OSA | | | | |
| --- | --- | --- | --- | --- |
| Effect type | **Effect** | **BOOT SE** | **Bootstrap 95%CI** | |
|  |  |  | **LLCI** | **ULCI** |
| Gross effect | 0.653 | 0.059 | 0.691 | 1.005 |
| Direct effect | 0.422 | 0.055 | 0.547 | 0.635 |
| Indirect effect | 0.231 | 0.021 | 0.01 | 0.15 |
| Mediation effect | **35.38%** |  |  |  |

Positive regression coefficients (β) indicate positive effects. Negative regression coefficients (β) indicate negative effects. ^*^P < 0.05 or the 95% bootstrap confidence interval does not straddle zero. Abbreviations: AHI: apnea hypoxia index, LPS: lipopolysaccharide, OSA: obstructive sleep apnea, SE: standard error, CI: confidence interval, LL: lower limit, UL: upper limit.

Table S3. Mediation analysis: the role of melatonin as a mediator of AHI-associated effect on the CRP level in patients with moderate-to-severe OSA.

| Regression analysis of mediation models (n=116) | | | | | | | | | | |
| --- | --- | --- | --- | --- | --- | --- | --- | --- | --- | --- |
| Variate | **Dependent variable: CRP** | |  | **Dependent variable: Melatonin** | |  | **Dependent variable:** **CRP** | | | |
|  | **β** | **t** |  | **β** | **t** |  | | **β** | **t** | |
| AHI | 0.226 | 3.544^*^ |  | -0.269 | -4.270^*^ |  | | 0.192 | 2.909^*^ | |
| Melatonin |  | |  |  | |  | | -0.128 | | -1.945 |
| R^2^ | 0.051 | |  | 0.073 | |  | | 0.066 | | |
| F | 12.560^*^ | |  | 18.232^*^ | |  | | 8.247^*^ | | |

| Melatonin mediated the effects of AHI on CRP level in patients with moderate-to-severe OSA | | | | |
| --- | --- | --- | --- | --- |
| Effect type | **Effect** | **BOOT SE** | **Bootstrap 95%CI** | |
|  |  |  | **LLCI** | **ULCI** |
| Gross effect | 0.0149 | 0.042 | 0.0066 | 0.0231 |
| Direct effect | 0.0126 | 0.043 | 0.0041 | 0.0211 |
| Indirect effect | 0.0023 | 0.014 | -0.001 | 0.0054 |
| Mediation effect | **/** |  |  |  |

Positive regression coefficients (β) indicate positive effects. Negative regression coefficients (β) indicate negative effects. ^*^P < 0.05 or the 95% bootstrap confidence interval does not straddle zero. Abbreviations: AHI: apnea hypoxia index, CRP: C-reactive protein, OSA: obstructive sleep apnea, SE: standard error, CI: confidence interval, LL: lower limit, UL: upper limit.

Table S4. Linear regression analysis, adjusted for age and BMI, showed that the relationships between sleep parameters, melatonin, intestinal barrier function biomarker (ZO-1) and inflammatory biomarkers (CRP and LPS) .

| Mild OSA | | | |  | Moderate OSA | | | |  | Severe OSA | | | |
| --- | --- | --- | --- | --- | --- | --- | --- | --- | --- | --- | --- | --- | --- |
| independent variables | dependent variables | β | P value |  | independent variables | dependent variables | β | P value |  | independent variables | dependent variables | β | P value |
| AHI | MT | -0.95 | ＜0.001 |  | AHI | MT | -0.965 | ＜0.001 |  | AHI | MT | -0.913 | ＜0.001 |
| LSpO2 |  | 0.097 | ＞0.05 |  | LSpO2 |  | 0.195 | ＞0.05 |  | LSpO2 |  | 0.285 | ＜0.05 |
| ODI |  | -0.534 | ＜0.001 |  | ODI |  | -0.659 | ＜0.001 |  | ODI |  | -0.758 | ＜0.001 |
| MAI |  | -0.188 | ＞0.05 |  | MAI |  | 0.015 | ＞0.05 |  | MAI |  | -0.339 | ＜0.01 |
|  |  |  |  |  |  |  |  |  |  |  |  |  |  |
| AHI | ZO-1 | 0.949 | ＜0.001 |  | AHI | ZO-1 | 0.958 | ＜0.001 |  | AHI | ZO-1 | 0.783 | ＜0.001 |
| LSpO2 |  | 0.065 | ＞0.05 |  | LSpO2 |  | -0.186 | ＞0.05 |  | LSpO2 |  | -0.37 | ＜0.01 |
| ODI |  | 0.443 | ＜0.05 |  | ODI |  | 0.515 | ＜0.001 |  | ODI |  | 0.625 | ＜0.001 |
| MAI |  | 0.285 | ＜0.05 |  | MAI |  | 0.07 | ＞0.05 |  | MAI |  | 0.209 | ＞0.05 |
|  |  |  |  |  |  |  |  |  |  |  |  |  |  |
| AHI | LPS | 0.203 | ＞0.05 |  | AHI | LPS | -0.078 | ＞0.05 |  | AHI | LPS | 0.346 | ＜0.05 |
| LSpO2 |  | -0.092 | ＞0.05 |  | LSpO2 |  | -0.236 | ＞0.05 |  | LSpO2 |  | -0.16 | ＞0.05 |
| ODI |  | 0.382 | ＜0.01 |  | ODI |  | -0.074 | ＞0.05 |  | ODI |  | 0.289 | ＞0.05 |
| MAI |  | -0.175 | ＞0.05 |  | MAI |  | -0.121 | ＞0.05 |  | MAI |  | 0.41 | ＜0.01 |
|  |  |  |  |  |  |  |  |  |  |  |  |  |  |
| AHI | CRP | 0.304 | ＜0.05 |  | AHI | CRP | 0.935 | ＜0.001 |  | AHI | CRP | 0.977 | ＜0.001 |
| LSpO2 |  | -0.05 | ＞0.05 |  | LSpO2 |  | -0.15 | ＞0.05 |  | LSpO2 |  | -0.145 | ＞0.05 |
| ODI |  | 0.338 | ＜0.01 |  | ODI |  | 0.664 | ＜0.001 |  | ODI |  | 0.515 | ＜0.01 |
| MAI |  | 0.304 | ＜0.05 |  | MAI |  | -0.025 | ＞0.05 |  | MAI |  | 0.396 | ＜0.01 |

OSA: obstructive sleep apnea, AHI: apnea-hypopnea index, LSpO2: lowest pulse oxygen saturation, ODI: oxygen desaturation index, MAI: micro-arousal index, MT: melatonin, ZO-1: zonula occludens-1, LPS: lipopolysaccharide, CRP: C-reactive protein.

Table S5. Mediation analysis: the role of melatonin as a mediator of AHI-associated effect on the ZO-1 level in patients with moderate-to-severe OSA, adjusted for age and BMI.

| Regression analysis of mediation models (n=116) | | | | | | | | | | |
| --- | --- | --- | --- | --- | --- | --- | --- | --- | --- | --- |
| Variate | **Dependent variable: ZO-1** | |  | **Dependent variable: Melatonin** | |  | **Dependent variable: ZO-1** | | | |
|  | **β** | **t** |  | **β** | **t** |  | | **β** | **t** | |
| AHI | 0.373 | 4.209^*^ |  | -0.422 | -4.873^*^ |  | | -0.014 | -0.190 | |
| Melatonin |  | |  |  | |  | | -0.751 | | -9.692^*^ |
| R^2^ | 0.189 | |  | 0.279 | |  | | 0.550 | | |
| F | 8.451^*^ | |  | 13.305^*^ | |  | | 30.286^*^ | | |

| Melatonin mediated the effects of AHI on ZO-1 level in patients with moderate-to-severe OSA | | | | |
| --- | --- | --- | --- | --- |
| Effect type | **Effect** | **BOOT SE** | **Bootstrap 95%CI** | |
|  |  |  | **LLCI** | **ULCI** |
| Gross effect | 2.7104 | 0.9095 | 0.9061 | 4.5148 |
| Direct effect | -0.1367 | 0.7177 | -1.5607 | 1.2873 |
| Indirect effect | 2.8471 | 0.6123 | 1.6149 | 4.0133 |
| Mediation effect | **/** |  |  |  |

Positive regression coefficients (β) indicate positive effects. Negative regression coefficients (β) indicate negative effects. ^*^P < 0.05 or the 95% bootstrap confidence interval does not straddle zero. Abbreviations: AHI: apnea hypoxia index, ZO-1: zonula occludens-1, OSA: obstructive sleep apnea, SE: standard error, CI: confidence interval, LL: lower limit, UL: upper limit.

Table S6. Mediation analysis: the role of melatonin as a mediator of AHI-associated effect on the LPS level in patients with moderate-to-severe OSA, adjusted for age and BMI.

| Regression analysis of mediation models (n=116) | | | | | | | | | | |
| --- | --- | --- | --- | --- | --- | --- | --- | --- | --- | --- |
| Variate | **Dependent variable: LPS** | |  | **Dependent variable: Melatonin** | |  | **Dependent variable: LPS** | | | |
|  | **β** | **t** |  | **β** | **t** |  | | **β** | **t** | |
| AHI | 0.198 | .040^*^ |  | -0.422 | -4.873^*^ |  | | 0.145 | 1.313 | |
| Melatonin |  | |  |  | |  | | -0.089 | | -0.786 |
| R^2^ | 0.058 | |  | 0.279 | |  | | 0.059 | | |
| F | 2.287^*^ | |  | 13.305^*^ | |  | | 1.607^*^ | | |

| Melatonin mediate the effects of AHI on LPS level in patients with moderate-to-severe OSA | | | | |
| --- | --- | --- | --- | --- |
| Effect type | **Effect** | **BOOT SE** | **Bootstrap 95%CI** | |
|  |  |  | **LLCI** | **ULCI** |
| Gross effect | 0.0154 | 0.0084 | -0.0012 | 0.0320 |
| Direct effect | 0.0122 | 0.0093 | -0.0062 | 0.0307 |
| Indirect effect | 0.0032 | 0.0041 | -0.0039 | 0.0118 |
| Mediation effect | **/** |  |  |  |

Positive regression coefficients (β) indicate positive effects. Negative regression coefficients (β) indicate negative effects. ^*^P < 0.05 or the 95% bootstrap confidence interval does not straddle zero. Abbreviations: AHI: apnea hypoxia index, LPS: lipopolysaccharide, OSA: obstructive sleep apnea, SE: standard error, CI: confidence interval, LL: lower limit, UL: upper limit.

Table S7. Mediation analysis: the role of melatonin as a mediator of AHI-associated effect on the CRP level in patients with moderate-to-severe OSA, adjusted for age and BMI.

| Regression analysis of mediation models (n=116) | | | | | | | | | | |
| --- | --- | --- | --- | --- | --- | --- | --- | --- | --- | --- |
| Variate | **Dependent variable: CRP** | |  | **Dependent variable: Melatonin** | |  | **Dependent variable:** **CRP** | | | |
|  | **β** | **t** |  | **β** | **t** |  | | **β** | **t** | |
| AHI | 0.217 | 2.147^*^ |  | -0.422 | -4.873^*^ |  | | -0.387 | -8.435^*^ | |
| Melatonin |  | |  |  | |  | | -1.080 | | -23.042* |
| R^2^ | 0.127 | |  | 0.279 | |  | | 0.877 | | |
| F | 4.395^*^ | |  | 13.305^*^ | |  | | 144.569^*^ | | |

| Melatonin mediated the effects of AHI on CRP level in patients with moderate-to-severe OSA | | | | |
| --- | --- | --- | --- | --- |
| Effect type | **Effect** | **BOOT SE** | **Bootstrap 95%CI** | |
|  |  |  | **LLCI** | **ULCI** |
| Gross effect | 0.0855 | 0.0778 | -0.0692 | 0.2403 |
| Direct effect | -0.2739 | 0.0325 | -0.3385 | -0.2093 |
| Indirect effect | 0.3594 | 0.0734 | 0.2115 | 0.5033 |
| Mediation effect | **/** |  |  |  |

Positive regression coefficients (β) indicate positive effects. Negative regression coefficients (β) indicate negative effects. ^*^P < 0.05 or the 95% bootstrap confidence interval does not straddle zero. Abbreviations: AHI: apnea hypoxia index, CRP: C-reactive protein, OSA: obstructive sleep apnea, SE: standard error, CI: confidence interval, LL: lower limit, UL: upper limit.
